# Supplementary material for: Polyphosphate acts as an architectural regulator of carbon fixation and nucleoid structure in cyanobacteria
Source: mBio. 2026 Aug 19;17(9):e00999-26. doi: 10.1128/mbio.00999-26 (PMC13556221; doi:10.1128/mbio.00999-26)
Supplement: Supplementary file 1 [file mbio.00999-26-s0001.docx]

**Figure S1. Quick-freeze deep-etch TEM images of *Synechococcus elongatus* PCC 7942.** Structures were labeled as carboxysomes ("C") if they exhibited an obvious protein shell, one or more flat faces, and/or densely packed Rubisco enzymes. Structures were labeled as polyphosphate granules ("P") if they were highly spherical and appeared amorphous. Log-phase cultures were quick-frozen at liquid-helium temperatures and processed for microscopy as described in Goodenough and Roth, 2025^60^. Micrographs courtesy of Ursula Goodenough and Robyn Roth.

**Figure S2 - PolyP granules remain nucleoid-associated after ciprofloxacin treatment. (A)** Microscopy images of WT exponential cells without (top row) and with ciprofloxacin treatment (bottom row), DAPI-stained nucleoid (magenta), polyP (cyan), and merge is the overlap of the 2 channels. Dotted white line represents the cell boundary from phase contrast (scale bar, 2 μm). **(B)** Pearson correlation coefficient of DAPI-stained nucleoid channel and polyP channel. Significance from Mann-Whitney. n = 3 biological replicates, bars show median and interquartile range (WT: n = 1720, Cipro: n = 428).

**Figure S3 - Nucleoid compaction in** Δ***mcdAB* cells can reduce carboxysome-polyP association. (A)** Microscopy images of WT exponential cells (top row) and Δ*mcdAB* cells (bottom row) after ciprofloxacin treatment. DAPI-stained nucleoid (magenta), polyP (cyan), carboxysomes (orange), overlay of polyP and carboxysome channels (cyan and orange) and merge is the overlap of all 3 channels. Dotted white line represents the cell boundary from phase contrast (scale bar, 2 μm). **(B)** Normalized Cross-Correlation Analysis (see description in Methods section) of carboxysomes and polyP granules from the same cell (top panel) and size-matched cell (bottom panel) for WT cells (left) (564 cells, 542 cells) and Δ*mcdAB* cells (right) (1904 cells, 1813 cells) after ciprofloxacin treatment. Color indicates the Pearson correlation coefficient (PCC), and the x- and y- axes demonstrate shifts of the carboxysome channel. The average PCC is reported below with the standard deviation.

**Figure S4 - Δ*ppk1* cells display altered cell morphology. (A)** Cell lengths for WT, Δ*ppk1*, Δ*ppk2*, Δ*ppx*, and Δ*ppk2*Δ*ppx* cells. **(B)** Cell widths for WT, Δ*ppk1*, Δ*ppk2*, Δ*ppx*, and Δ*ppk2*Δ*ppx* cells. **(C)** Cell areas for WT, Δ*ppk1*, Δ*ppk2*, Δ*ppx*, and Δ*ppk2*Δ*ppx* cells. All plots display significance from one-way ANOVA and Dunnett’s multiple comparisons. n = 3 biological replicates, bars show median and interquartile range (WT (2312 cells), Δ*ppk1* (873 cells), Δ*ppk2* (2442 cells), Δ*ppx* (885 cells), and Δ*ppk2*Δ*ppx* (963 cells)).

**VIDEO LEGENDS**

**Video 1. Carboxysome dynamics in polyP metabolism mutants of *S. elongatus***. Phase contrast channel overlaid with carboxysomes (RbcS-mTQ, LUT is orange) in the following strains - WT (far left), Δ*ppk1* (left), Δ*ppk2* (center), Δ*ppx* (right), Δ*ppk2*Δ*ppx* (right).One-hour video at 1 frame every 3 minutes. Playback speed is 10 frames per second (scale bar is 2 μm).

**SUPPLEMENTARY TABLES**

**Supplementary Table 1 -** Python Analysis Settings

| **Focus Detected** | **Background Subtraction** | **Image Denoise** | **Add Gaussian Blur** | **Detection** |
| --- | --- | --- | --- | --- |
| RbcS-mOrg Carboxysomes | restoration.rolling_ball(cell_fluor,radius =  3) | filters.unsharp_mask(cell_fluor_bg, radius  = 2, amount = 5) | filter.gaussian(cell_fluor_sharp, 0.01) | min_sigma = 2.75, max_sigma = 3,  threshold = 0.005, overlap = 0.75 |
| RbcS-mTQ Carboxysomes | restoration.rolling_ball(cell_fluor,radius =  3) | filters.unsharp_mask(cell_fluor_bg, radius  = 2, amount = 5) | filter.gaussian(cell_fluor_sharp, 0.01) | min_sigma = 1.75, max_sigma = 3,  threshold = 0.0025, overlap = 0.75 |
| polyP | restoration.rolling_ball(cell_fluor,radius =  3) | filters.unsharp_mask(cell_fluor_bg, radius  = 3, amount = 5) | filters.gaussian(cell_fluor_sharp, .5) | min_sigma = 2.5, max_sigma = 5,  threshold = 0.0125, overlap = 0.95 |

**Supplementary Table 2 -** Constructs used

| **Strain** | **Identifier** | **Description** | **Reference** |
| --- | --- | --- | --- |
| WT | C1 | WT *Synechococcus elongatus* PCC 7942 |  |
| Δ*ccm* | C103 | mNG-McdB + RbcS-mTQ + ΔccmK2-O | ^20^ |
| Δ*mcdA* | C7 | Native deletion of mcdA by replacing the gene with a kanamycin resistance cassette | ^20^ |
| Δ*mcdB* | C8 | Native deletion of mcdB by replacing the gene with a kanamycin resistance cassette | ^20^ |
| Δ*mcdAB* | C9 | Native deletion of mcdAB by replacing the operon with a kanamycin resistance cassette | ^20^ |
| RbcS-mTQ | C3 | RbcS-mTQ with the native promoter at neutral site I | ^20^ |
| RbcS-mOrange2 | C30 | RbcS-mOrange2 with the native promoter at neutral site I | ^20^ |
| Δ*mcdA* + RbcS-mOrange2 | C107/CED28 | Native deletion of mcdA by replacing the gene with a kanamycin resistance cassette, RbcS-mOrange2 with the native promoter at neutral site I | This paper |
| Δ*mcdB* + RbcS-mOrange2 | C108/CED29 | Native deletion of mcdB by replacing the gene with a kanamycin resistance cassette, RbcS-mOrange2 with the native promoter at neutral site I | This paper |
| Δ*mcdAB* + RbcS-mOrange2 | C109/CED30 | Native deletion of mcdAB by replacing the operon with a kanamycin resistance cassette, RbcS-mOrange2 with the native promoter at neutral site I | This paper |
| Δ*ppk1* | C66/CED11 | Native deletion of ppk1 by replacing the gene with a spectinomycin resistance cassette | This paper |
| Δ*ppk1* + RbcS-mTQ | C69/CED14 | Native deletion of ppk1 by replacing the gene with a spectinomycin resistance cassette, RbcS-mTQ with the native promoter at neutral site I | This paper |
| Δ*ppk1* + RbcS-mOrange2 | C110/CED31 | Native deletion of ppk1 by replacing the gene with a spectinomycin resistance cassette, RbcS-mOrg with the native promoter at neutral site I | This paper |
| Δ*ppk2* | C122/CED42 | Native deletion of ppk2 by replacing the gene with a kanamycin resistance cassette | This paper |
| Δ*ppk2* + RbcS-mTQ | C125/CED46 | Native deletion of ppk2 by replacing the gene with a kanamycin resistance cassette, RbcS-mTQ with the native promoter at neutral site I | This paper |
| Δ*ppk2* + RbcS-mOrange2 | C127/CED48 | Native deletion of ppk2 by replacing the gene with a kanamycin resistance cassette, RbcS-mOrg with the native promoter at neutral site I | This paper |
| Δ*ppx* | C73/CED18 | Native deletion of ppx by replacing the gene with a spectinomycin resistance cassette | This paper |
| Δ*ppx* + RbcS-mTQ | C76/CED21 | Native deletion of ppx by replacing the gene with a spectinomycin resistance cassette, RbcS-mTQ with the native promoter at neutral site I | This paper |
| Δ*ppx* + RbcS-mOrange2 | C111/CED32 | Native deletion of ppx by replacing the gene with a spectinomycin resistance cassette, RbcS-mOrg with the native promoter at neutral site I | This paper |
| Δ*ppx +* Δ*ppk2* | C124/CED45 | Native deletion of ppx by replacing the gene with a spectinomycin resistance cassette, native deletion of ppk2 by replacing the gene with a kanamycin resistance cassette | This paper |
